# Supplementary material for: A Fluoroponytailed NHC–Silver Complex Formed from Vinylimidazolium/AgNO3 under Aqueous–Ammoniacal Conditions
Source: Molecules. 2022 Jun 28;27(13):4137. doi: 10.3390/molecules27134137 (PMC9268170; doi:10.3390/molecules27134137)
Supplement: Supplementary file 1 [file molecules-27-04137-s001.zip › molecules-1736720-supplementary.pdf]

Supporting Information

A Fluoroponytailed NHC- Silver Complex formed from Vinyl-imidazolium /$AgNO_3$ under Aqueous-Ammoniacal Conditions

Gabriel Partl [1], Marcus Rauter [2], Lukas Fliri [3], Thomas Gelbrich [4], Christoph Kreutz [5], Thomas Müller [5], Volker Kahlenberg [6], Sven Nerdinger [7*] and Herwig Schottenberger [1*]

[1] Institute of General, Inorganic and Theoretical Chemistry, University of Innsbruck, CCB, Innrain 80-82, 6020 Innsbruck, Austria; gabriel.j.partl@gmail.com; herwig.schottenberger@uibk.ac.at
[2] Infineon Technologies AG, Siemensstrasse 2, 9500 Villach, Austria; marcus.rauter@infineon.com
[3] Aalto University, Department of Bioproducts and Biosystems, Post Office Box 16300, 00076 Aalto, Finland; lukas.fliri@aalto.fi
[4] Institute of Pharmacy, Leopold Franzens University of Innsbruck, Innrain 52c, 6020 Innsbruck, Austria; thomas.gelbrich@uibk.ac.at
[5] Institute Organic Chemistry, University of Innsbruck, CCB, Innrain 80-82, 6020 Innsbruck, Austria; thomas.mueller@uibk.ac.at; Christoph.kreutz@uibk.ac.at
[6] Institute of Mineralogy & Petrography, University of Innsbruck, Innrain 52, 6020 Innsbruck, Austria; volker.kahlenberg@uibk.ac.at
[7] Sandoz GmbH, Biochemiestr. 10, 6250 Kundl, Austria; sven.nerdinger@sandoz.com
* Correspondence: sven.nerdinger@sandoz.com; herwig.schottenberger@uibk.ac.at
† Dedicated to the memory of Professor Victor Snieckus.

Content

Figure S1: ^{13}C NMR spectrum (176 MHz, acetone-d_6) of [bis(3-(1H,1H,2H,2H-perfluoroctyl)-1-vinylimidazol-2-ylidene)silver(I)] nitrate (**Ag(FNHC)$_2$NO$_3$**)........ 3

Figure S2: ^1H NMR spectrum (300 MHz, acetone-d_6) of **Ag(FNHC)$_2$NO$_3$**........ 3

Figure S3: ^{19}F NMR spectrum (659 MHz, acetone-d_6) of **Ag(FNHC)$_2$NO$_3$**........ 4

Figure S4: ^{19}F - ^{13}C HSQC NMR spectrum (^{19}F frequency =659 MHz, acetone-d_6) of **Ag(FNHC)$_2$NO$_3$**. ^{13}C (^{19}F decoupled) trace is shown on the left; ^{19}F trace is shown on top. 4

Figure S5: ATR-FT-IR spectrum (neat) of **Ag(FNHC)$_2$NO$_3$**. 5

Figure S6: Plot showing Ag-C bond lengths (x-axis) and their corresponding C-Ag-C bond angles (y-axis) in reported bis-carbene-Ag complexes in comparison to those of **Ag(FNHC)$_2$NO$_3$**. 6

Figure S7: Powder diffractogram of **Ag(FNHC)$_2$NO$_3$** (top) and comparison to simulated powder diffraction pattern (bottom). 7

Figure S8: High resolution mass spectrum of **Ag$^+$(FNHC)$_2$** (top) and simulated spectrum thereof (bottom). The peak at 441.0610 m/z is attributable to the 3-(1H,1H,2H,2H-perfluoroctyl)-1-vinylimidazolium ion. 7

Figure S9: High resolution mass spectrum of the molecule ion peak **Ag$^+$(FNHC)$_2$** (top) and simulated spectrum thereof (bottom). 8

Elemental analysis........ 8

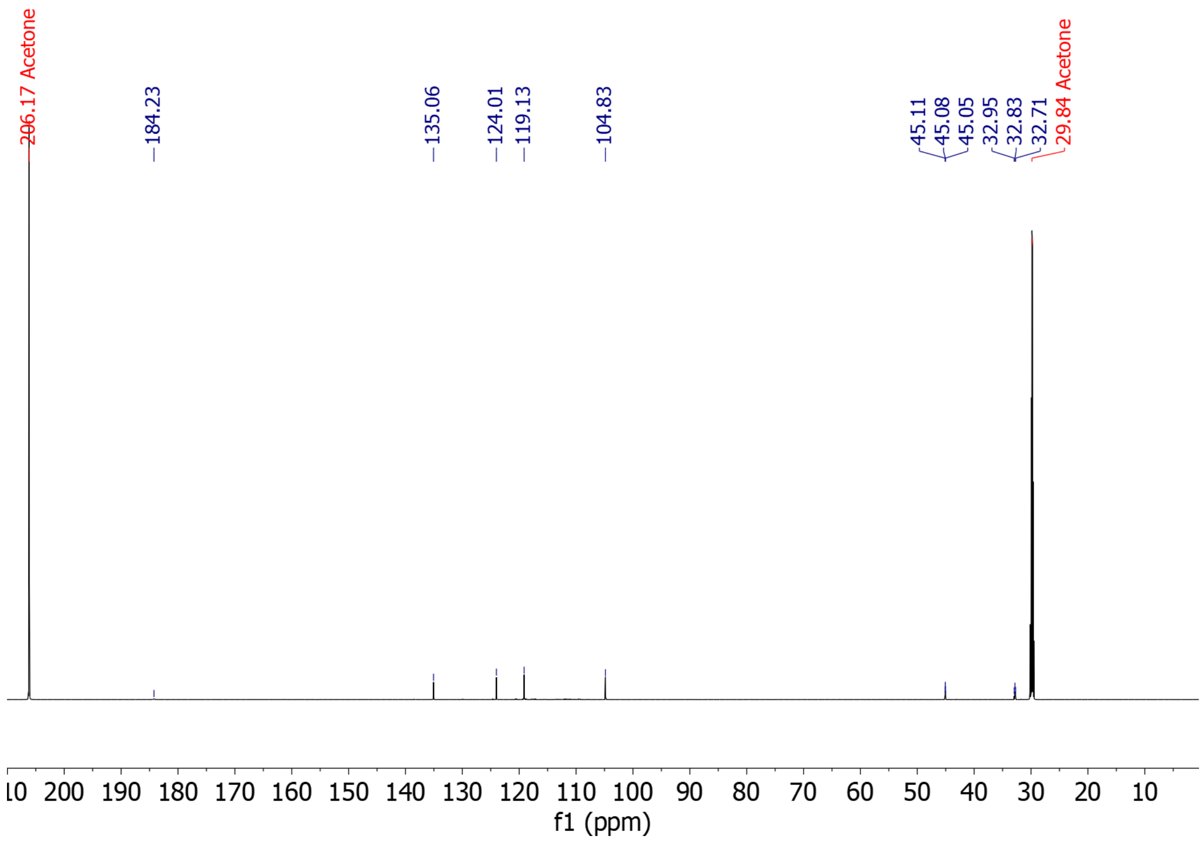

Figure S1: ^{13}C NMR spectrum (176 MHz, acetone-d_6) of [bis(3-(1*H*,1*H*,2*H*,2*H*-perfluoroctyl)-1-vinylimidazol-2-ylidene)silver(I)] nitrate (**Ag(FNHC)$_2$NO$_3$**).

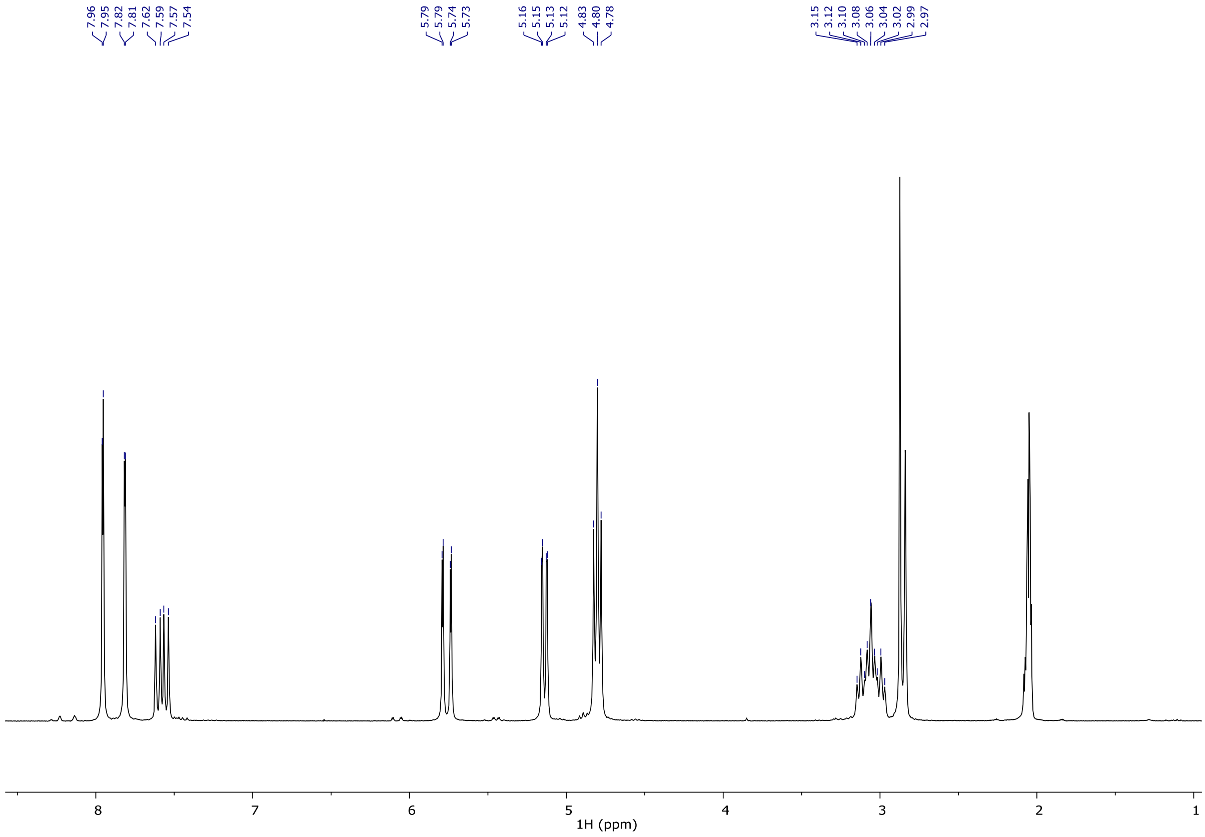

Figure S2: ^{1}H NMR spectrum (300 MHz, acetone-d_6) of **Ag(FNHC)$_2$NO$_3$**.

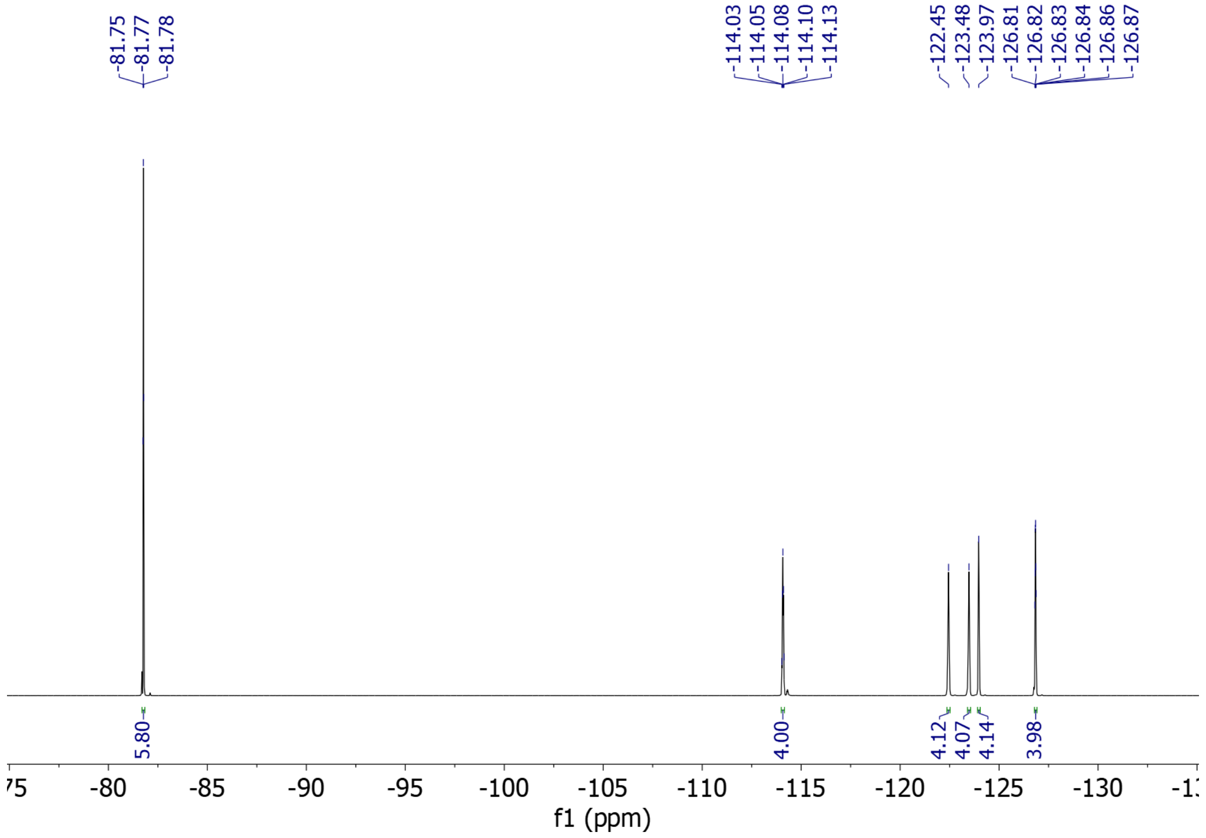

Figure S3: ^{19}F NMR spectrum (659 MHz, acetone-d_6) of **Ag(FNHC)$_2$NO$_3$**.

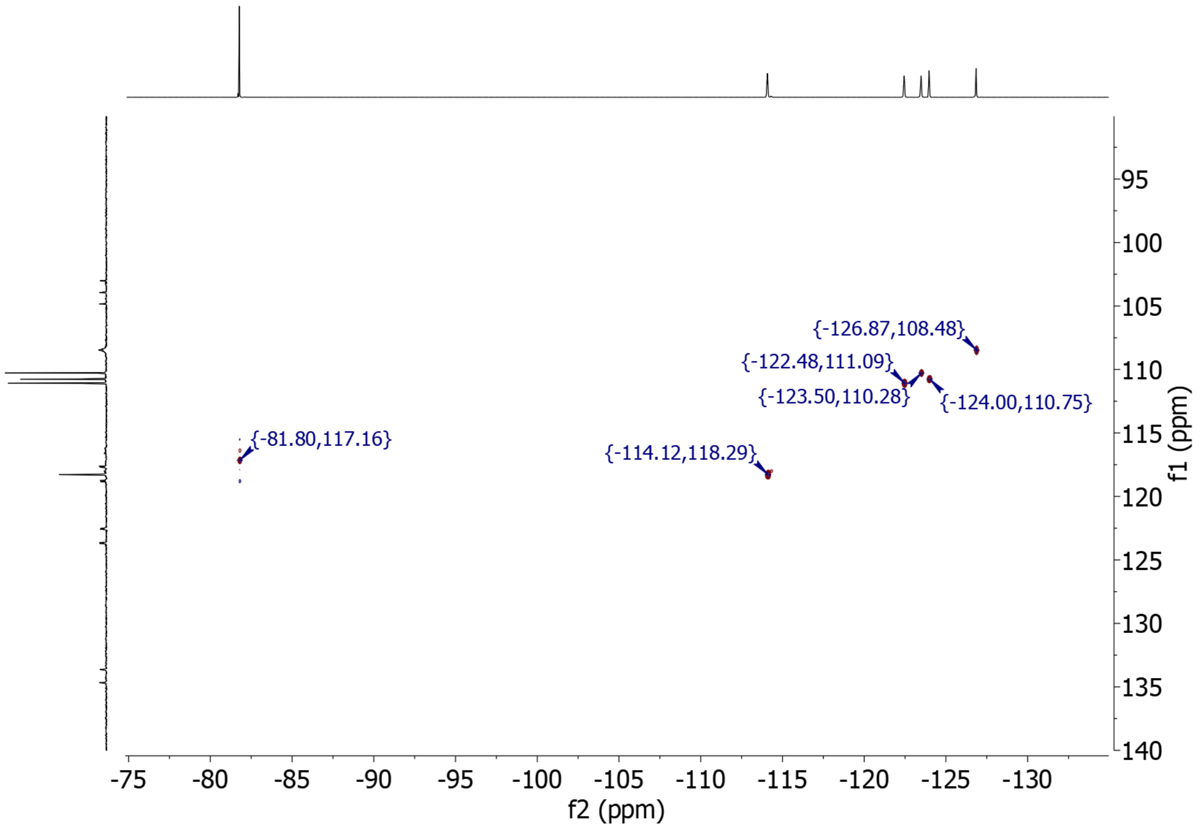

s

Figure S4: ^{19}F - ^{13}C HSQC NMR spectrum (^{19}F frequency =659 MHz, acetone-d_6) of **Ag(FNHC)$_2$NO$_3$**. ^{13}C (^{19}F decoupled) trace is shown on the left; ^{19}F trace is shown on top.

Note: For clarity and better s/n ratio, the ^{13}C trace shown in Figure **S4** is ^{19}F decoupled. The ^{13}C trace in Figure **S1** is ^{1}H decoupled and thus looks markedly different.

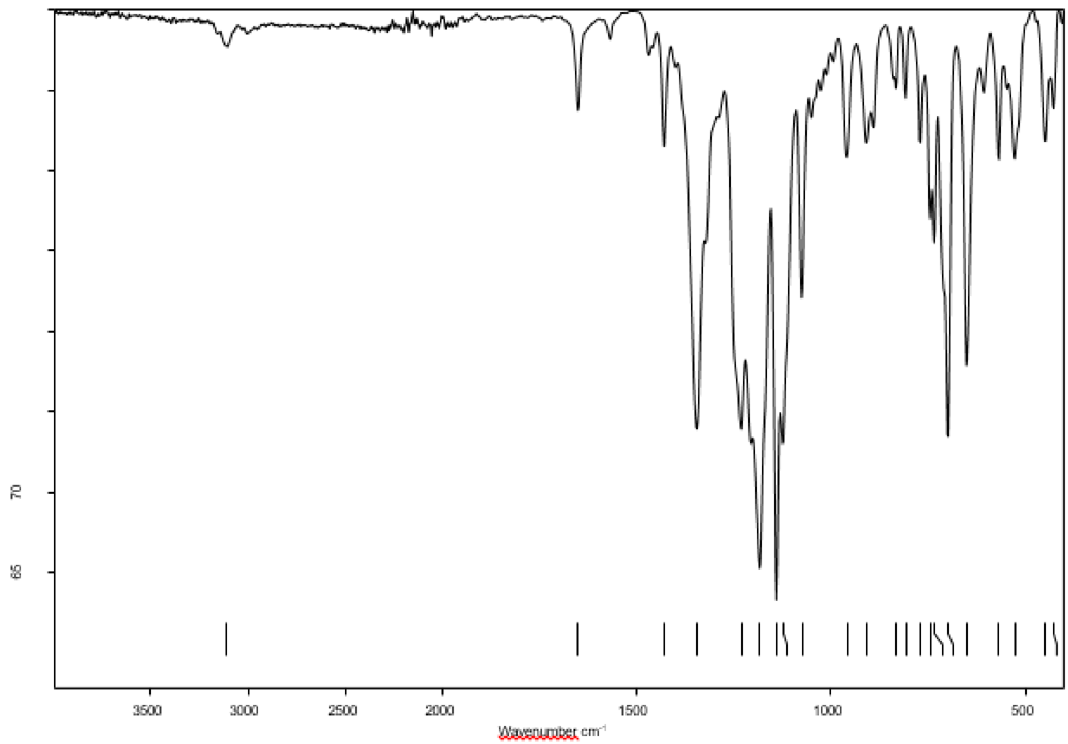

Figure S5: ATR-FT-IR spectrum (neat) of **Ag(FNHC)$_2$NO$_3$**.

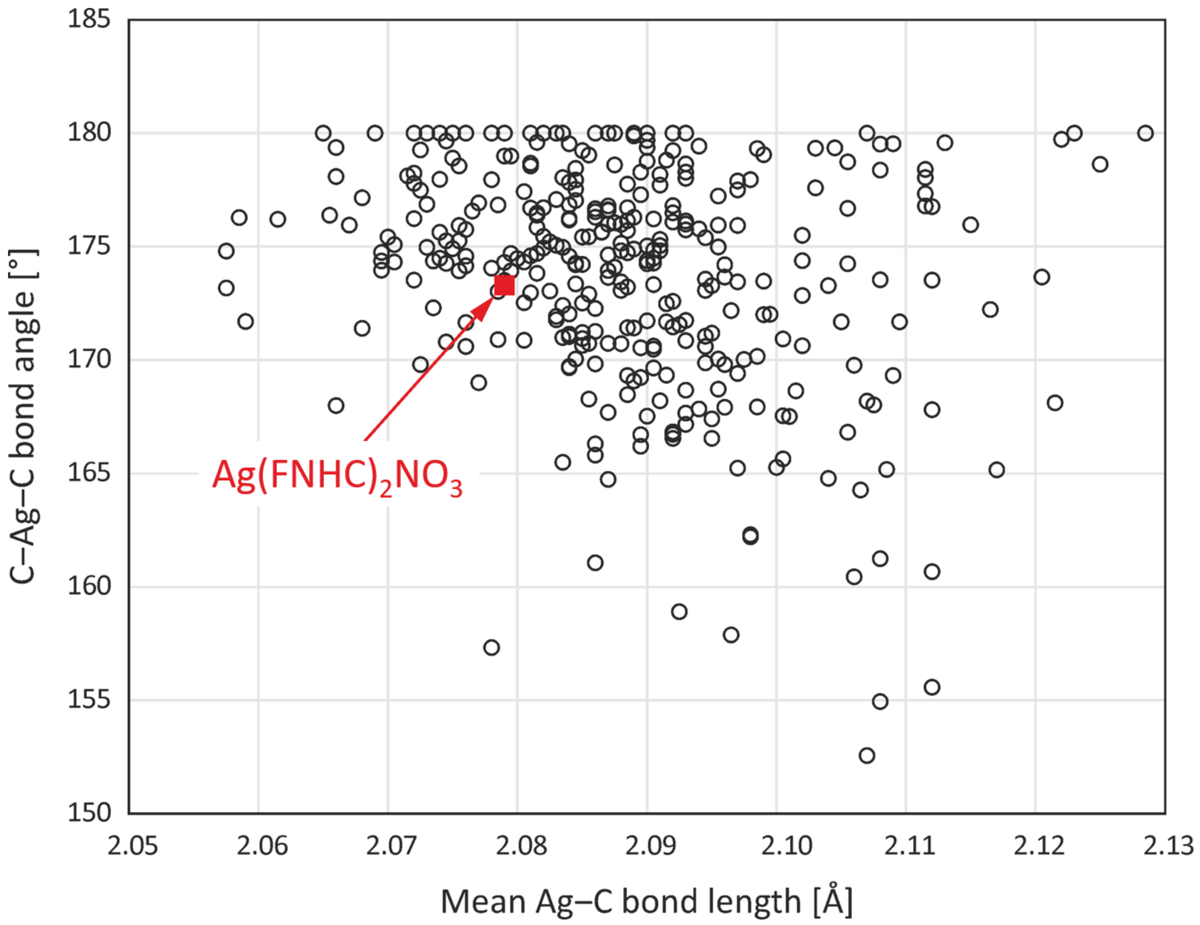

Figure S6: Plot showing Ag-C bond lengths (x-axis) and their corresponding C-Ag-C bond angles (y-axis) in reported bis-carbene-Ag complexes in comparison to those of **Ag(FNHC)$_2$NO$_3$**.

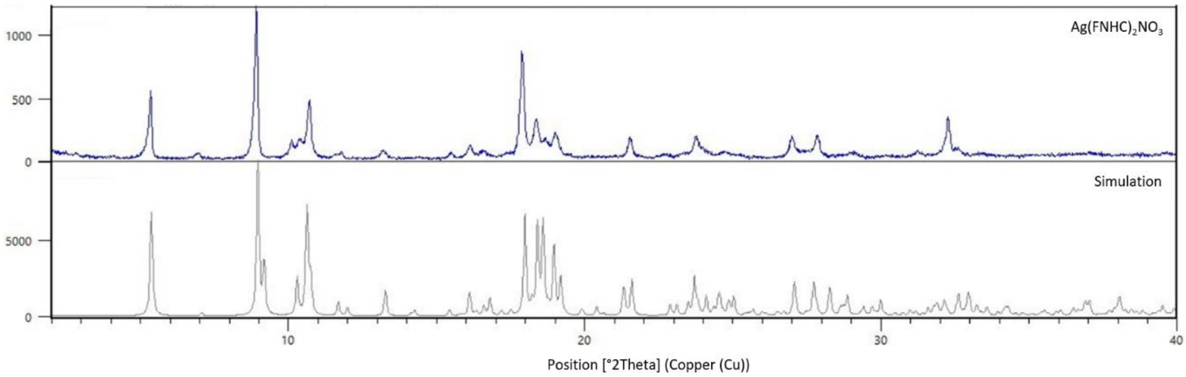

Figure S7: Powder diffractogram of **Ag(FNHC)$_2$NO$_3$** (top) and comparison to simulated powder diffraction pattern (bottom).

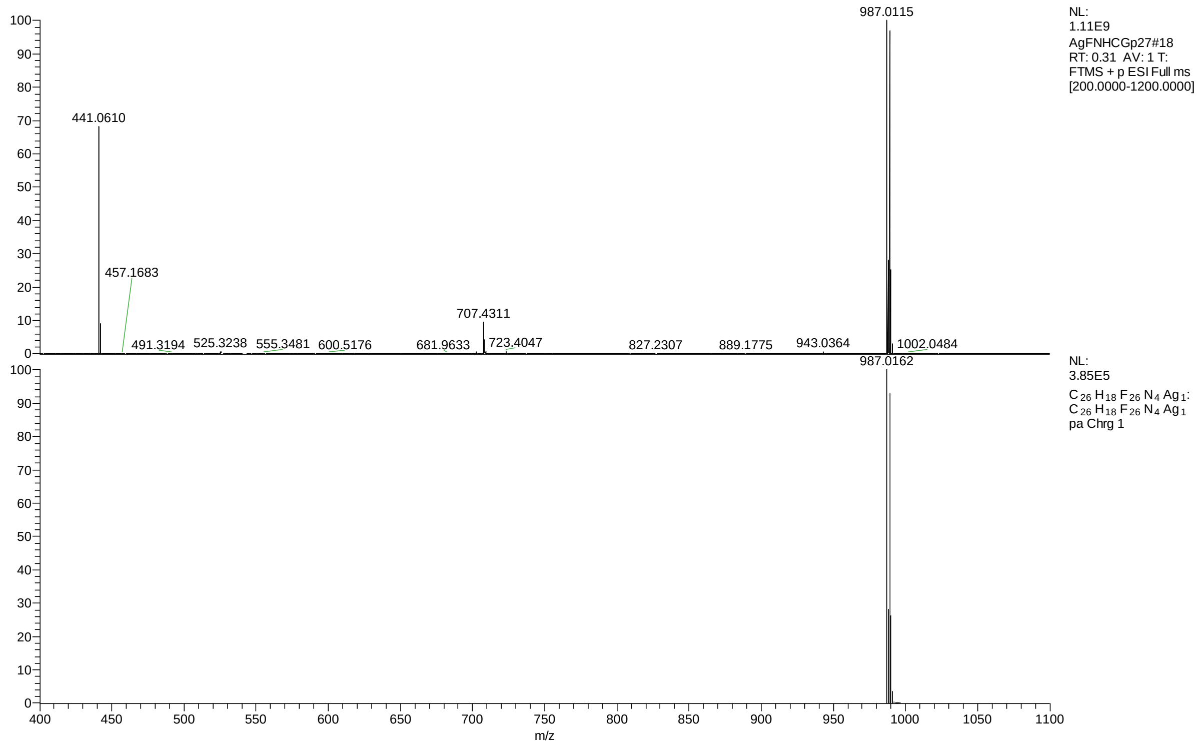

Figure S8: High resolution mass spectrum of **Ag$^+$(FNHC)$_2$** (top) and simulated spectrum thereof (bottom). The peak at 441.0610 m/z is attributable to the 3-(1*H*,1*H*,2*H*,2*H*-perfluoroctyl)-1-vinylimidazolium ion.

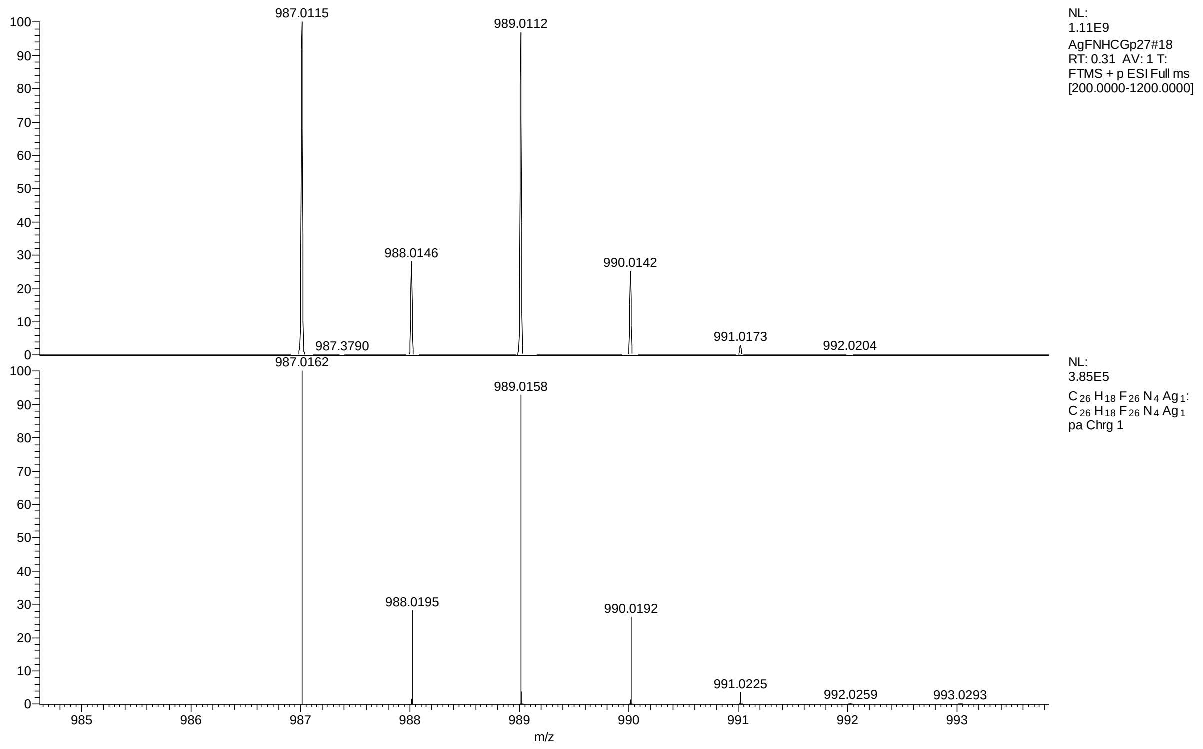

Figure S9: High resolution mass spectrum of the molecule ion peak **$Ag^{+}(FNHC)_2$** (top) and simulated spectrum thereof (bottom).

Elemental analysis

Elemental composition values for $C_{26}H_{18}AgF_{26}N_5O_3$	Calculated	Found
C	29.73 %	28.61%
H	1.73 %	1.55%
N	6.67%	6.36%

Note: Composition values for carbon and hydrogen likely deviate from the expected values due to reluctant combustion behavior of the polyfluorinated compound.
